# *Lactobacillus paracasei* subsp. *paracasei* 2004 improves health and lifespan in *Caenorhabditis elegans*

**DOI:** 10.1038/s41598-024-60580-y

**Published:** 2024-05-07

**Authors:** Saya Kishimoto, Masanori Nono, Yutaka Makizaki, Yoshiki Tanaka, Hiroshi Ohno, Eisuke Nishida, Masaharu Uno

**Affiliations:** 1 RIKEN Center for Biosystems Dynamics Research (BDR), 2-2-3 Minatojima-Minamimachi, Chuo-Ku, Kobe, 650-0047 Japan; 2 R&D Center, Biofermin Pharmaceutical Co. Ltd., 7-3-4 Ibukidai-Higashimachi, Nishi-Ku, Kobe, 651-2242 Japan

**Keywords:** *Caenorhabditis elegans*, Lactic acid bacterium (LAB), Health, Longevity, The IIS pathway, Genetics, Gene expression

## Abstract

Recent research has highlighted the importance of the gut microbiome in regulating aging, and probiotics are interventions that can promote gut health. In this study, we surveyed several novel lactic acid bacteria to examine their beneficial effect on organismal health and lifespan in *C. elegans*. We found that animals fed some lactic acid bacteria, including *L. acidophilus* 1244 and *L. paracasei* subsp. *paracasei* 2004, grew healthy. Supplementation with the lactic acid bacterial strains *L. acidophilus* 1244 or *L. paracasei* subsp. *paracasei* 2004 significantly improved health, including food consumption, motility, and resistance to oxidative stressor, hydrogen peroxide. Our RNA-seq analysis showed that supplementation with *L. paracasei* subsp. *paracasei* 2004 significantly increased the expression of *daf-16*, a *C. elegans* FoxO homolog, as well as genes related to the stress response. Furthermore, *daf-16* deletion inhibited the longevity effect of *L. paracasei* subsp. *paracasei* 2004 supplementation. Our results suggest that *L. paracasei* subsp. *paracasei* 2004 improves health and lifespan in a DAF-16-dependent manner.

## Introduction

Recent research has revealed the crucial role that the human microbiome plays in regulating the aging process^1^. Studies have shown that changes in the composition of the microbiome can influence age-related decline in organ function and the onset of age-related diseases, such as Alzheimer’s disease and cardiovascular disease^2–10^. The microbiome supports the immune system, aids in digestion and metabolism, and helps prevent colonization by pathogenic microorganisms^11^. These functions are essential in older adults because they could help delay age-related changes in the body and improve overall health^12–15^. Therefore, understanding the role of the microbiome in aging regulation is critical for developing interventions that can promote healthy aging and prevent age-related diseases.

The diversity of the gut microbiome is established early in life and is shaped by a variety of factors: birth and early life, diet, antibiotic use, age, geography, and lifestyle^12–15^. As individuals age, the diversity of the gut microbiota tends to decrease, with certain bacteria becoming more abundant while others decline^12,14^. This shift in microbial composition has been associated with alterations in gut permeability, inflammation, and immune function, which may contribute to the development of age-related diseases^2,6–8,10^. For example, some studies have found that the gut microbiota of elderly individuals is more pro-inflammatory and less diverse than that of younger individuals^12–15^ and that these changes are associated with an increased risk of conditions such as cardiovascular disease and dementia^2,8^. In *Drosophila*, transplantation of the microbiota from aged donor flies to young recipient flies changes the microbiota and decreases lifespan^16^. Conversely, transplantation of the microbiome from young fish to middle-aged fish alters the microbiome composition of the older recipient and significantly increases the lifespan in killifish^17^. Fecal microbiome transplantation from wild-type to progeria mice recipients enhances health and lifespan^18^. These lines of evidence suggest a crucial role of the microbiota in lifespan regulation.

Keeping the gut microbiome in good condition could contribute to health and longevity. Interventions to maintain the gut microbiome in good condition are a matter of interest. Probiotics are live microorganisms that are administered to promote gut health adequately: probiotics represent an essential group of beneficial consumed microorganisms found in fermented foods such as yogurt, kefir, and sauerkraut, as well as in supplemental forms^19,20^. Administration of the probiotic *Lactobacillus* GKM3 promotes longevity and memory retention in SAMP8 mice, a model of accelerated aging^21^. Supplementation with *Akkermansia muciniphila* improves insulin sensitivity and reduces insulinemia and plasma total cholesterol levels in volunteer humans^22^. Some probiotics have been shown to increase lifespan in *Caenorhabditis elegans*: lactic acid bacteria (*Lactobacillus gasseri* SBT2055 and *Lactobacillus rhamnosus* CNCM I-3690) and non-lactic acid bacteria (*Propionibacterium freudenreichii*) increase the lifespan of *C. elegans*^23–25^. Other probiotics might be beneficial for improving health and longevity.

In this study, we surveyed several lactic acid bacteria to find their beneficial effects on health and lifespan. To this end, *C. elegans*, one of the most used model organisms in aging research, was fed with several lactic acid bacteria after their development, and we examine the effect of lactic acid bacteria administration on growth, health, and longevity.

## Results

**Figure 1:**
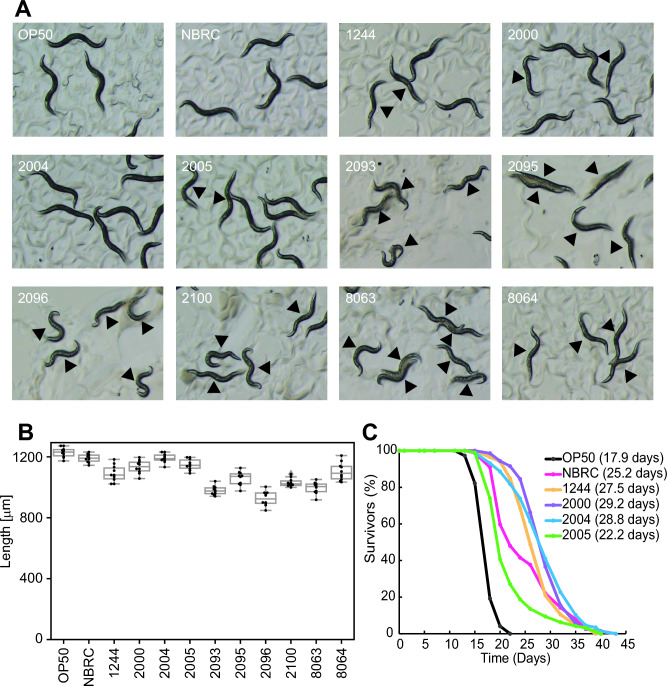
*L. acidophilus* 1244 and *L. paracasei* subsp. *paracasei* 2004 supplementation extends lifespan. (**A**) Representative images of wild-type animals fed the indicated bacterium for 3 days from Day 1 of adulthood. Feeding some lactic acid bacterial strains (*L. acidophilus* 1244, *L. gasseri* 2000*,* 2100*,* 8063*, and* 8064, *L. paracasei* subsp. *paracasei* 2004 and 2005, *L. gasseri* 2093, and *L. johnsonii* 2095 and 2096) lead to bags of worms, one of the egg-laying defects. Arrowheads indicate animals showing bags of worms. (**B**) The body length of animals fed the indicated bacterium on Day 4 of adulthood. Feeding lactic acid bacterial strains except for *L. rhamnosus* CNCM I-3690 and *L. paracasei* subsp. *paracasei* 2004 shortened the body compared with *E. coli* OP50 feeding. See Table S1 for detailed statistics. (**C**) Survival curves of animals fed the indicated bacterial strains. Feeding lactic acid bacterial strains extended the lifespan compared with *E. coli* OP50 feeding. See Table S2 for detailed statistics.

To identify the lactic acid bacterial strain that positively regulates the lifespan of *C. elegans*, we first examined the effect of lactic acid bacterial strains on the growth of the animals. Age-synchronized germ-free populations can be obtained by treating animals with bleach. We focused on analyzing the beneficial effects of lactic acid bacteria on health and longevity rather than on their impact on development. Therefore, Day1 adult animals (after completion of development: 3 days after synchronization) were fed lactic acid bacterial strains (*L. acidophilus* 1244, *L. gasseri* 2000*,* 2100*,* 8063*,* and 8064, *L. paracasei* subsp. *Paracasei* 2004 and 2005, *L. gasseri* 2093, and *L. johnsonii* 2095 and 2096), including the lactic acid bacterial strain *L. rhamnosus* CNCM I-3690, which has been shown to extend the lifespan of wild-type animals^25^, for 3 days, and the length of the animals was measured. Animals fed lactic acid bacterial strains except for *L. rhamnosus* CNCM I-3690 and *L. paracasei* subsp. *Paracasei* 2004 were significantly shorter than animals fed *E. coli* OP50; animals fed *L. rhamnosus* CNCM I-3690 or *L. paracasei* subsp. *Paracasei* 2004 was not significantly shorter (Fig. 1A, B and Table S1). Furthermore, animals grew almost normally when fed some lactic acid bacteria (*L. rhamnosus* CNCM I-3690, *L. acidophilus* 1244, *L. gasseri* 2000, and *L. paracasei* subsp. *Paracasei* 2004 and 2005), while most animals displayed bags of worms (state of animals where many fertilized eggs, normally laid outside, overly remained in the germline, one of the egg-laying defects^26^; Fig. 1A, arrowhead) when fed other lactic acid bacteria (*L. gasseri* 2100*,* 8063, and 8064*, L. gasseri* 2093, and *L. johnsonii* 2095 and 2096). Bags of worms are induced when animals are subjected to harsh environments such as fasting^27^. We thus focused on the lactic acid bacterial strains that did not induce egg-laying defects and examined their effect on lifespan. Our measurements showed that animals fed lactic acid bacterial strains (*L. acidophilus* 1244, *L. gasseri* 2000, and *L. paracasei* subsp. *paracasei* 2004 and *2005*) as well as the *L. rhamnosus* CNCM I-3690 strain lived significantly longer than animals fed control food (*E. coli* OP50) (Fig. 1C and Table S2). These results suggest that some lactic acid bacterial strains have the potential to positively regulate lifespan.

**Figure 2:**
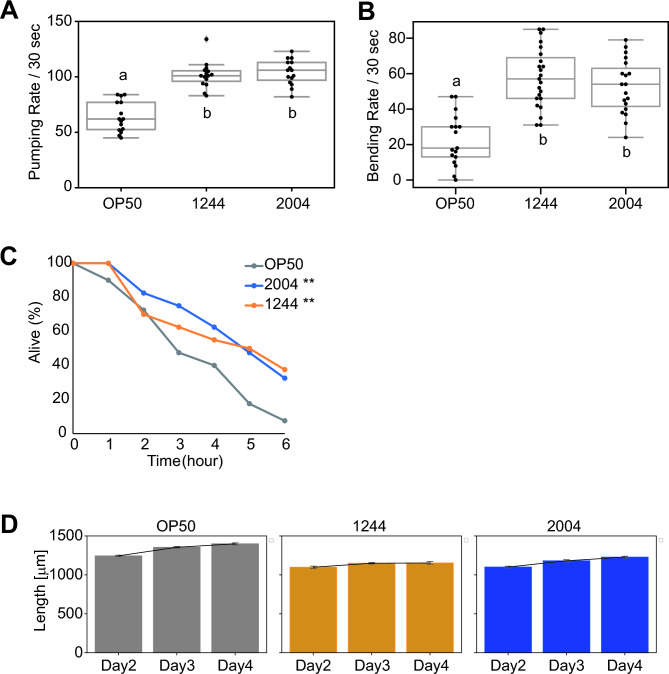
*L. acidophilus* 1244 and *L. paracasei* subsp. *paracasei* 2004 feeding enhances health. (**A**) The pumping rate of the animals fed the indicated bacterium on Day 4 of adulthood. *L. acidophilus* 1244 and *L. paracasei* subsp. *paracasei* 2004 feeding improved the pumping rate at middle age. Means not sharing the same letter are significantly different (Tukey test, *p* < 0.05). See Table S3 for detailed statistics. (**B**) The bending rate of the animals fed the indicated bacterium on Day 10. *L. acidophilus* 1244 and *L. paracasei* subsp. *paracasei* 2004 feeding improved the bending rate at middle age. Means not sharing the same letter are significantly different (Tukey test, *p* < 0.05). See Table S4 for detailed statistics. (**C**) The survival curves of the animals fed the indicated bacterium on Day 10 in 2 mM hydrogen peroxide. *L. acidophilus* 1244 and *L. paracasei* subsp. *paracasei* 2004 feeding improved the oxidative stress resistance at middle age. *P*-values were calculated by a log-rank test with Bonferroni correction (**, *p* < 0.01). See Table S5 for detailed statistics. (**D**) Body size of the animals fed the indicated bacterium on Day 2, Day 3, and Day 4 of adulthood. *L. acidophilus* 1244 and *L. paracasei* subsp. *paracasei* 2004 feeding increased body size on Day 4. See Table S6 for detailed statistics.

We focused on *L. acidophilus* 1244 and *L. paracasei* subsp. *paracasei* 2004, both of which extended the lifespan (Fig. 1C and Table S2), because *L. acidophilus* 1244 and *L. paracasei* subsp. *paracasei* 2004 had the strongest and the weakest effect on body size, respectively, among the strains that extended lifespan (Fig. 1B and Table S1). We then examined the effect of these lactic acid bacterial strains on health as well as lifespan. To this end, animals were fed lactic acid bacteria for 3 days from Day 1 of adulthood and then we examined the pumping rate, the bending rate, and oxidative stress resistance, all of which are indicators of health^28^, using middle-aged animals. Our measurements showed that *L. acidophilus* 1244 and *L. paracasei* subsp. *paracasei* 2004 feeding significantly increased the pumping rate (Fig. 2A and Table S3), the bending rate (Fig. 2B and Table S4), and oxidative stress resistance (Fig. 2C and Table S5) compared with *E. coli* OP50 feeding. These results indicate that *L. acidophilus* 1244 and *L. paracasei* subsp. *paracasei* 2004 supplementation improves the animals’ health as well as lifespan.

Because both *L. acidophilus* 1244 and *L. paracasei* subsp. *paracasei* 2004 feeding affects growth (Fig. 1B and Table S1), it is possible that supplementation with these two lactic acid bacteria causes dietary restriction, which promotes health and lifespan. We thus scrutinized whether *L. acidophilus* 1244 and *L. paracasei* subsp. *paracasei* 2004 feeding could mimic food restriction because of the avoidance of these bacteria or malnutrition under the use of these bacteria as a food resource. We examined the body size every day after lactic acid bacterium feeding. Animals fed *L. acidophilus* 1244 stopped growing on Day 3 of adulthood, while animals fed OP50 or *L. paracasei* subsp. *paracasei* 2004 continued growing on Day 4, although the size of animals fed *L. paracasei* subsp. *paracasei* 2004 was smaller than that of the animals fed *E. coli* OP50 (Fig. 2D and Table S6). This result implies that *L. acidophilus* 1244 feeding causes malnutrition that makes it difficult for animals to grow, while *L. paracasei* subsp. *paracasei* 2004 is nutritious enough to allow the animals to grow steadily.

**Figure 3:**
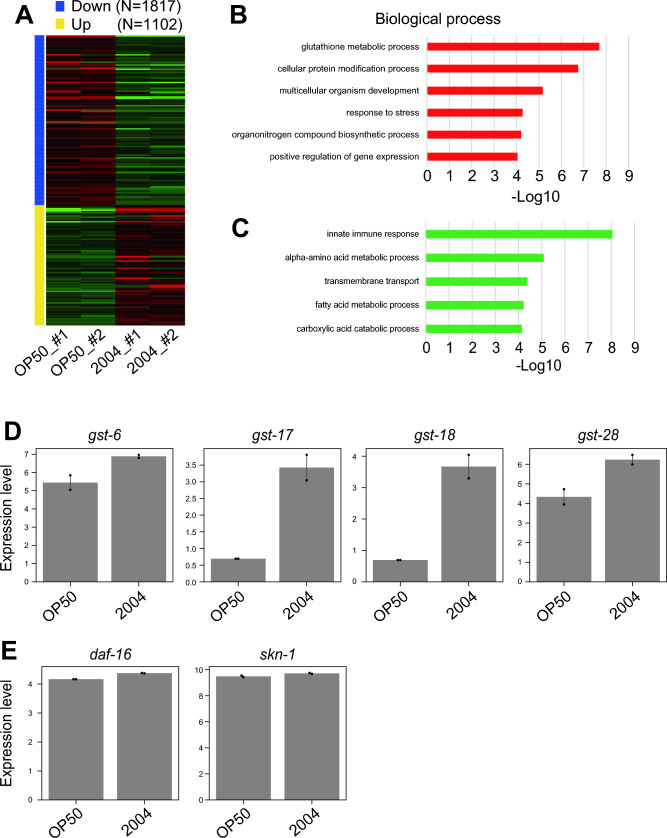
*L. paracasei* subsp. *paracasei* 2004 feeding upregulates the expression of genes related to stress response. (**A**) Heatmap of differentially expressed genes (DEGs) in response to *L. paracasei* subsp. *paracasei* 2004 feeding (FDR cutoff < 0.1, minimum fold change = 2.0). *L. paracasei* subsp. *paracasei* 2004 feeding upregulates 1102 genes and downregulates 1817 genes. (**B**, **C**) Gene Ontology (GO) analysis of biological processes: the 1102 (> 2.0-fold, FDR < 0.1) upregulated gene list (**B**) and the 1817 (< 0.5-fold, FDR < 0.1) downregulated gene list were analyzed by PANTHER^32^. GO terms corresponding to biological processes whose p-value was less than 0.0001 were extracted. (**D**, **E**) Bar plots show the expression level of glutathione genes (**D**) and transcription factors, *daf-16* and *skn-1* (**E**).

Our results show that *L. paracasei* subsp. *paracasei* 2004 feeding, compared with *L. acidophilus* 1244 feeding, has a more prominent effect on lifespan (Fig. 1C and Table S2) and a weaker food restriction effect on body size (Fig. 2D and Table S6). Transcription factors, such as DAF-16 and SKN-1, regulate longevity in *C. elegans*, and transcriptome alterations are vital for lifespan regulation^29–31^. Therefore, we then explored the effect of *L. paracasei* subsp. *paracasei* 2004 feeding on the transcriptome profile to understand the mechanisms of the longevity effect of lactic acid bacteria. We explored the transcriptome alterations after 3 days of *L. paracasei* subsp. *paracasei* 2004 feeding by RNA-seq analysis. We defined the differentially expressed genes (DEGs) whose fold changes were more than twofold with an FDR cutoff (less than 0.1). There were 1102 upregulated DEGs and 1817 downregulated DEGs in response to *L. paracasei* subsp. *paracasei* 2004 feeding (Fig. 3A). Gene Ontology analysis with PANTHER^32^ revealed that genes related to glutathione metabolic process, cellular protein modification process, multicellular organism development, and response to stress were overrepresented among the upregulated DEGs (Fig. 3B); genes related to innate immune response, alpha-amino acid metabolic process, transmembrane transport, fatty acid process, and carboxylic acid catabolic process were overrepresented among the downregulated DEGs (Fig. 3B–E). Glutathione plays a vital role in the antioxidative process^33^, which is consistent with our observation that *L. paracasei* subsp. *paracasei* 2004 feeding improved oxidative stress resistance (Fig. 2C and Table S5).

**Figure 4:**
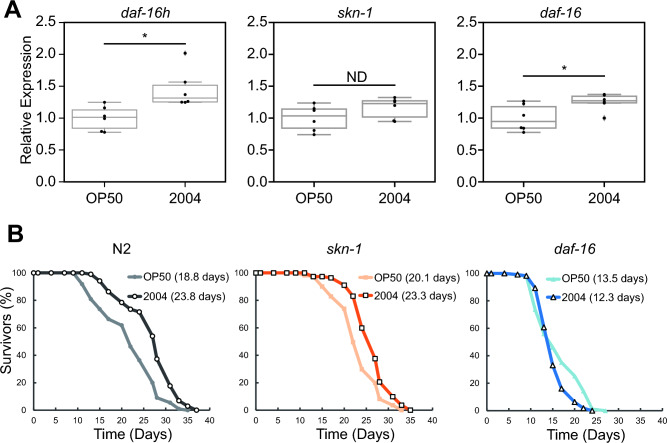
Longevity increase induced by *L. paracasei* subsp. *paracasei* 2004 feeding requires the transcription factor DAF-16. (**A**) Box plot shows the relative mRNA levels of *daf-16 h* (left), *skn-1* (middle), and *daf-16* (right) in animals fed the indicated bacterium. *L. paracasei* subsp. *paracasei* 2004 feeding upregulated the expression of *daf-16* mRNA. P-values were calculated by an unpaired t-test. *, *p* < 0.05. (**B**) Survival curves of the wild-type N2 (left) and two mutants, EU31[*skn-1(zu135)*] and CF1038[*daf-16(mu86)*], fed the indicated bacterium. *L. paracasei* subsp. *paracasei* 2004 feeding extended the *C. elegans* lifespan in a DAF-16-dependent manner. *P*-values were calculated by a log-rank test with Bonferroni correction. **, *p* < 0.01. See Table S8 for detailed statistics.

The upregulated DEGs included *daf-16 h*, one of the *daf-16* transcript variants, and *skn-1* (Fig. 3E and Table S6), both of which are transcription factors promoting stress resistance and longevity in *C. elegans*^29,30^*.* This suggests that lactic acid bacterial strain *L. paracasei* subsp. *paracasei* 2004 feeding may induce these transcription factors and play an essential role in longevity. Thus, we validated the upregulation of the *daf-16 h* and *skn-1* genes by lactic acid bacterial strain *L. paracasei* subsp. *paracasei* 2004 feeding. Our analyses indicated that the expression of *daf-16* and *daf-16 h* was slightly upregulated with statistical significance while that of *skn-1* was not (Fig. 4A). These results suggest that DAF-16 might be involved in *L. paracasei* subsp. *paracasei* 2004 feeding-induced longevity. To examine this possibility, we measured the lifespan of wild-type N2 and *skn-1(zu135)* and *daf-16(mu86)* deletion mutants fed *L. paracasei* subsp. *paracasei* 2004 (Fig. 4B). While *L. paracasei* subsp. *paracasei* 2004 feeding significantly increased the lifespan of wild-type animals, and that of the *skn-1* deletion mutant, *daf-16* deletion suppressed the longevity effect induced by *L. paracasei* subsp. *paracasei* 2004 feeding. These results suggest that DAF-16 plays a vital role in lifespan extension by *L. paracasei* subsp. *paracasei* 2004 feeding.

## Discussion

Our study aimed to investigate the potential effect of various lactic acid bacteria on health and longevity. We found that administration of *L. acidophilus* 1244 and *L. paracasei* subsp. *paracasei* 2004 has a longevity effect. Interestingly, short-term supplementation of these bacteria in early life (for 3 days from Day 1 of adulthood) significantly improved health, including food consumption, motility rate, and resistance to oxidative stress in middle-aged animals. These results suggest that supplementation with these bacteria ameliorates the fitness decline with age. Animals fed *L. paracasei* subsp. *paracasei* 2004 continued to grow, indicating that supplementation with this bacterial strain could have a beneficial probiotic effect on the host organism, *C. elegans*. On the other hand, we observed that feeding *L. acidophilus* 1244 resulted in stunted growth. Because some probiotics improve health^34^ and lifespan^35^ through mimicking dietary restriction, *L. acidophilus* 1244 administration might also improve health and lifespan by mimicking the dietary restriction. Further study should be done to examine mechanisms underlying improved health and lifespan by *L. acidophilus* 1244 administration.

The proposed pro-longevity mechanisms of probiotics include modulating immune responses and stress responses^20^. Supplementation of some lactic acid bacterial strains has been shown to enhance longevity in *C. elegans* through the transcription factors DAF-16 and SKN-1, both of which play a role in pro-longevity effects through modulating immune responses and stress responses^23,25,36–38^. Our study demonstrated that the longevity increase induced by *L. paracasei* subsp. *paracasei* 2004 supplementation requires DAF-16, but not SKN-1, and that administration with this bacterial strain increases the expression of genes related to stress responses but decreases the expression of genes related to the innate immune system. These results suggest that *L. paracasei* subsp. *paracasei 2004* might increase lifespan by enhancing stress responses through the activity of DAF-16.

Overall, we found that the lactic acid bacterial strain *L. paracasei* subsp. *paracasei* 2004 can potentially improve health and lifespan with one-time supplementation for 3 days in *C. elegans*. The mechanism underlying this effect of the bacterial strain on healthy aging is mediated by DAF-16-induced stress response modulation, a highly conserved pro-longevity transcription factor. The involvement of DAF-16 in the beneficial effects of *L. paracasei* subsp. *paracasei* 2004 suggests potential applications in other organisms. Further research is needed to fully understand these mechanisms and assess their relevance in other organisms.

## Methods

### Bacterial strains and culture conditions

*E. coli* OP50 was provided by the Caenorhabditis Genetics Center, University of Minnesota (CGC), and used as a control food source. *E. coli* OP50 was grown in LB medium at 37 °C for 10–12 h with shaking. Lactic acid bacterial strains were grown at 37 °C in a modified GAM medium (29.5 g of Nissui modified GAM broth, 3.5 g of glucose, 0.5 g of tween80 in 500 mL of H_2_O) without shaking for one or two days until the lactic acid bacteria grew. Bacteria were harvested by centrifugation at 3,000 × g for 10 min and washed three times: twice in M9 buffer and then once in S basal buffer. Then, the bacteria were adjusted to a final concentration of 5 × 10^9^ CFU (colony-forming units).

### Nematodes and growth conditions

The *C. elegans* Bristol strain N2 and mutant strains EU31[*skn-1(zu135)*] and CF1038[*daf-16(mu86)*] were provided by the CGC. The Bristol N2 strain was used for all measurements except those in the longevity assay using the EU31[*skn-1(zu135)*] and CF1038[*daf-16(mu86)*] mutants. Animals were maintained on nematode growth medium (NGM) plates seeded with *E. coli* OP50, as explained before^39^ or lactic acid bacterial strains. We defined animals as Day 1 adult animals 3 days after hatching. Synchronized germ-free animals were obtained by the bleaching method^40^.

### Body size measurements

Synchronized eggs were grown on NGM plates seeded with *E. coli* OP50 until animals reached adulthood. Then, the animals were transferred to M9 buffer containing *E. coli* OP50 or lactic acid bacterial strains. The animals were incubated with gentle shaking. After 3 days, images of the animals were taken on Day 4 of adulthood with a stereomicroscope (Olympus SZX16). Images were analyzed using ImageJ software.

### Longevity assay

For Fig. 1C, trials were conducted on NGM plates containing 5-fluoro-2′-deoxyuridine (FUdR) at 100 μg/mL seeded with *E. coli* OP50 or lactic acid strains from Day 1 of adulthood until all animals died. For Fig. 4B, young adult (Day 1) animals (3 days after synchronization) were cultured in *E. coli* OP50 or *L. paracasei* subsp. *paracasei* 2004 suspended in S basal buffer with FUdR (100 μg/mL). After 3 days, the animals were transferred, and trials were conducted on NGM plates with FUdR (100 μg/mL) seeded with *E. coli* OP50. Mortality was scored every 2 or 3 days when the animals were transferred to a new plate. Animals were scored as dead if they failed to respond to being touched by a pick. Survival plots were generated by using single lifespan data.

### Pharyngeal pumping rate measurements

For pharyngeal pumping rate measurements, Day 1 adulthood animals were cultured on NGM plates containing FUdR seeded with *E. coli* OP50, *L. acidophilus* 1244*,* or *L. paracasei* subsp. *paracasei* 2004 for 3 days. Animals’ pumping rate on food was recorded. The number of pumping events was measured under an optical microscope for 30 s. One pharyngeal pump was defined as a complete forward and backward movement of the grinder in the pharynx.

### Bending rate measurements

For bending measurements, Day 1 adult animals (3 days after synchronization) were transferred to NGM plates containing FUdR seeded with *E. coli* OP50, *L. acidophilus* 1244*,* or *L. paracasei* subsp. *paracasei* 2004 for 3 days. After Day 4 of adulthood, animals were transferred and cultured on NGM plates seeded with *E. coli* OP50 until Day 10 of adulthood. Then, the animals’ bending rate in M9 buffer was recorded under an optical microscope (Olympus SZX16 with CellSens system) for 30 s. The number of bends was counted manually afterward.

### Oxidative stress assay

For the oxidative stress assay, Day 1 adult animals were transferred to NGM plates containing FUdR seeded with *E. coli* OP50, *L. acidophilus* 1244*,* or *L. paracasei* subsp. *paracasei* 2004 for 3 days. After Day 4 of adulthood, animals were transferred and cultured on NGM plates seeded with *E. coli* OP50 until Day 10 of adulthood, and they were cultured for nine days on NGM plates seeded with *E. coli* OP50. Then, the animals were soaked in M9 buffer with 2 mM hydrogen peroxide. The animals’ movements in response to blue light were recorded hourly under the camera system (DMK23GP031, ImageingSource) for one minute. The movement of each animal was measured using Python (code used for analyses is provided in the Supplementary manuscript). Animals were scored as dead if they failed to move in response to blue light for two hours in a row. The oxidative stress assays were repeated twice, and survival plots were generated using single lifespan data.

### RNA-seq analysis

Total RNA was isolated using TRIzol reagent (Invitrogen) from around 200 *C. elegans* Bristol strain N2 cultured on NGM plates with *E. coli* OP50 or *L. paracasei* subsp. *paracasei* 2004 for 3 days from Day 1 of adulthood. RNA-seq library preparation and RNA sequencing were performed at Macrogen Inc. using the Illumina HiSeq 2000 platform. FASTQC^41^ was used to inspect the quality scores of the raw sequence data and to look for biases. The reads were trimmed using the Cutadapt^42^ wrapper, TrimGalore^43^. The reads were mapped by aligning the software HISAT2^44^ to the reference genome (WB235). The mapped reads were sorted by SAMtools^45^. Read counts per gene were obtained using Stringtie^46^. The DEGs obtained from RNA-seq-based expression profiling were analyzed by using iDEP0.96 (Integrated Differential Expression and Pathway analysis) online tools^47^. GO analysis on DEGs was performed using PANTHER^32^: statistical overrepresentation test of up-regulated or down-regulated DEGs was conducted with an option, biological GO terms. Expression level is log_2_(CPM: counts per million + 2).

### Quantitative RT‒PCR

Total RNA was isolated using TRIzol reagent (Invitrogen) from around 200 *C. elegans* Bristol strain N2 cultured on NGM plates with *E. coli* OP50 or *L. paracasei* subsp. *paracasei 2004* with FUDR supplementation for 3 days from Day 1 of adulthood. The isolated total RNA was reverse transcribed into single-stranded cDNA using ReverTra Ace qPCR RT Master Mix with gDNA remover (TOYOBO) according to the manufacturer’s protocol. Quantitative RT‒PCR was performed with an ABI 7300 Real-Time PCR system (Applied Biosystem) using Power SYBR® Green Master Mix (Thermo Fisher Scientific). Relative mRNA quantification was performed with the standard curve method. The relative mRNA levels were normalized to the expression of *act-1*, a *C. elegans* housekeeping gene. Primer sequences were determined using Primer3Plus (https://www.primer3plus.com/), and we used the primers that did not generate non-specific products or primer dimers, which was verified with the melt curve analysis.

- *act-1* Fw: 5′- CCCATCAACCATGAAGATCAA-3′
- *act-1* Rv: 5′-CACATCTGTTGGAAGGTGGA-3′
- *daf-16 h* Fw: 5′-TTCTCACAGGACATGCAAGC-3′
- *daf-16 h* Rv: 5′-ACGCTCTTGTTGATGGAGGT-3′
- *daf-16* Fw: 5′-TGGAATTCAATCGTGTGGAA-3′
- *daf-16* Rv: 5′-ATGAATATGCTGCCCTCCAG-3′
- *skn-1* Fw: 5′-CTCCATTCGGTAGAGGACCA-3′
- *skn-1* Rv: 5′-ACTGATCAGCAGGAGCCACT-3′

### Supplementary Information

Supplementary Information 1.

Supplementary Tables.

## Data Availability

The RNA sequencing data generated during the current study are available in the NCBI Sequence Read Archive (Accession#: GSE241495). All the other datasets are available from the corresponding author upon reasonable request.
