# Supplementary material for: Lactobacillus paracasei subsp. paracasei 2004 improves health and lifespan in Caenorhabditis elegans
Source: Sci Rep. 2024 May 7;14:10453. doi: 10.1038/s41598-024-60580-y (PMC11076489; doi:10.1038/s41598-024-60580-y)
Supplement: Supplementary file 1 — Supplementary Information 1. [file 41598_2024_60580_MOESM1_ESM.pdf]

```python
import numpy as np
import cv2
import os, glob

arr_dir = "./array_data"
bi_dir = "./binary_data"
res_dir = "./result_data"

movies = glob.glob("./*.avi")

"""
!!!!!!!!!! Set [time, x_grid, y_grid, well_size, threshold] !!!!!!!!!!!
"""
time = 5
well_size = 63
x_grid = 403
y_grid = 40
threshold = 30

def movie2array(movie, time, well_size, x_grid, y_grid):
    """
    transform movie into gray movie
    """
    cap = cv2.VideoCapture(movie)
    fps = cap.get(cv2.CAP_PROP_FPS)
    n_frames = int(fps * time) + 1
    frames = []
    while True:
        ret, frame = cap.read()
        gray = cv2.cvtColor(frame, cv2.COLOR_BGR2GRAY)
        gray = np.array(gray, dtype="float64") / 255
        if not ret:
            print("read error")
            break
        frames.append(gray)
        if len(frames) > n_frames:
            break
    if cap.isOpened():
```

```
        cap.release()
    """
    crop the movie to analyze
    """
    cropped_frames = []
    lis = np.arange(n_frames)
    for i in lis:
        cropped_frames.append(frames[i][y_grid: y_grid+(well_size*12), x_grid:
x_grid+(well_size*20)])
    np.array(cropped_frames)
    return cropped_frames, n_frames

def array2binary(cropped_frames, n_frames, threshold):
    """
    calcuate mean_frame
    """
    lis = np.arange(n_frames)
    frame = cropped_frames
    mean_frame = np.zeros(frame[0].shape)
    for i in lis:
        mean_frame += frame[i]
    mean_frame /= n_frames
    """
    subtract mean_frame
    """
    edited_frames = []
    for j in lis:
        arr = frame[j] - mean_frame
        arr[arr < 0] = 0
        edited_frames.append(arr)
    """
    make binary image
    """
    for k in lis:
        edited_frames[k][edited_frames[k] < float(threshold)/255] = 0
        edited_frames[k][edited_frames[k] >= float(threshold)/255] = 1
    np.array(edited_frames)
    return edited_frames
```

```python
def binary2result(edited_frames, n_frames, well_size):
    """
    make different frames
    """
    binary = edited_frames
    diff_frames = []
    for bef, aft in zip(binary[0:(n_frames-1)], binary[1:n_frames]):
        diff_frame = aft - bef
        diff_frame[diff_frame < 0] = 0
        diff_frames.append(diff_frame)
    """
    calculate sum of the frames
    """
    lis = np.arange(n_frames-1)
    total = np.zeros(binary[0].shape)
    for i in lis:
        total += diff_frames[i]
    np.array(total)
    result = np.zeros((12,20))
    yn = np.arange(12)
    xn = np.arange(20)
    for y in yn:
        for x in xn:
            Y = int(well_size * (y + 0.5))
            X = int(well_size * (x + 0.5))
            result[y, x] = total[(Y-20): (Y+20), (X-20): (X+20)].sum()
    return total, result

for i, movie in enumerate(movies):
    if __name__ == '__main__':
        # run movie2array function
        cropped_frames, n_frames = movie2array(movie, time, well_size, x_grid, y_grid)
        """
        # save cropped_frames in array_data folder
        np.save(arr_dir + "/array_{0}_1".format(i), cropped_frames)
        print("movie2array {0} done".format(i))
        """
```

```python
# run array2binary function
edited_frames = array2binary(cropped_frames, n_frames, threshold)
"""
# save edited_frames in binary_data folder
np.save(bi_dir + "/binary_{0}_1".format(i), edited_frames)
print("array2binary {0} done".format(i))
"""
# run binary2result function
total, result = binary2result(edited_frames, n_frames, well_size)
# save result in result_data folder
total_cv = np.array(total / (n_frames-1) * 255, dtype='int')
cv2.imwrite(res_dir + "/sum_{0}_1.png".format(i), total_cv)
np.save(res_dir + "/sum_{0}_1".format(i), total)
np.savetxt(res_dir + "/result_{0}_1.csv".format(i), result, delimiter=",")
print("Movie {0} done".format(i))
```

```python
import numpy as np
import cv2
import os, glob
from scipy import ndimage

arr_dir = "./array_data"
bi_dir = "./binary_data"
res_dir = "./result_data"

movies = glob.glob("./*.avi")
"""
!!!!!!!!!! Set [time, x_grid, y_grid, well_size, threshold, sigma] !!!!!!!!!!!
"""
time = 1 # duration of time analyzed
well_size = 63 # well size (pixels) in a movie
x_grid = 403 # starting point (x) of the ROI
y_grid = 40 # starting point (y) of the ROI
threshold = 30 # a threshold value to make the binary image
sigma = 1 # a sigma value for gaussian filter

def movie2array(movie, time, well_size, x_grid, y_grid):
    """
    transform movie into gray movie
    """
    cap = cv2.VideoCapture(movie)
    fps = cap.get(cv2.CAP_PROP_FPS)
    n_frames = int(fps * time) + 1
    frames = []
    while True:
        ret, frame = cap.read()
        gray = cv2.cvtColor(frame, cv2.COLOR_BGR2GRAY)
        gray = np.array(gray, dtype="float64") / 255
        if not ret:
            print("read error")
            break
        frames.append(gray)
        if len(frames) > n_frames:
            break
```

```
    if cap.isOpened():
        cap.release()
    """
    crop the movie to analyze
    """
    cropped_frames = []
    lis = np.arange(n_frames)
    for i in lis:
        cropped_frames.append(frames[i][y_grid: y_grid+(well_size*12), x_grid: 
x_grid+(well_size*20)])
    np.array(cropped_frames)
    return cropped_frames, n_frames

def array2binary(cropped_frames, n_frames, threshold, sigma):
    """
    calcuate mean_frame
    """
    lis = np.arange(n_frames)
    frame = cropped_frames
    mean_frame = np.zeros(frame[0].shape)
    for i in lis:
        mean_frame += frame[i]
    mean_frame /= n_frames
    """
    subtract mean_frame
    """
    edited_frames = []
    for j in lis:
        arr = frame[j] - mean_frame
        arr[arr < 0] = 0
        arr = ndimage.gaussian_filter(arr, sigma)
        edited_frames.append(arr)
    """
    make binary image
    """
    for k in lis:
        edited_frames[k][edited_frames[k] < float(threshold)/255] = 0
        edited_frames[k][edited_frames[k] >= float(threshold)/255] = 1
```

```
    np.array(edited_frames)
    return edited_frames

def binary2result(edited_frames, n_frames, well_size):
    """
    make different frames
    """
    binary = edited_frames
    diff_frames = []
    for bef, aft in zip(binary[0:(n_frames-1)], binary[1:n_frames]):
        diff_frame = aft - bef
        diff_frame[diff_frame < 0] = 0
        diff_frames.append(diff_frame)
    """
    calculate sum of the frames
    """
    lis = np.arange(n_frames-1)
    total = np.zeros(binary[0].shape)
    for i in lis:
        total += diff_frames[i]
    np.array(total)
    result = np.zeros((12,20))
    yn = np.arange(12)
    xn = np.arange(20)
    for y in yn:
        for x in xn:
            Y = int(well_size * (y + 0.5))
            X = int(well_size * (x + 0.5))
            result[y, x] = total[(Y-20): (Y+20), (X-20): (X+20)].sum()
    return total, result

for i, movie in enumerate(movies):
    if __name__ == '__main__':
        # run movie2array function
        cropped_frames, n_frames = movie2array(movie, time, well_size, x_grid, y_grid)
        """
        # save cropped_frames in array_data folder
        np.save(arr_dir + "/array_{0}_2".format(i), cropped_frames)
```

```
print("movie2array {0} done".format(i))
"""
# run array2binary function
edited_frames = array2binary(cropped_frames, n_frames, threshold, sigma)
"""
# save edited_frames in binary_data folder
np.save(bi_dir + "/binary_{0}_2".format(i), edited_frames)
print("array2binary {0} done".format(i))
"""
# run binary2result function
total, result = binary2result(edited_frames, n_frames, well_size)
# save result in result_data folder
total_cv = np.array(total / (n_frames-1) * 255, dtype='int')
cv2.imwrite(res_dir + "/sum_{0}_2.png".format(i), total_cv)
np.save(res_dir + "/sum_{0}_2".format(i), total)
np.savetxt(res_dir + "/result_{0}_2.csv".format(i), result, delimiter=",")
print("movie {0} done".format(i))
```

```python
import pandas as pd
import numpy as np
from pandas import DataFrame
import glob


tidy_dir = "./result_data/tidy_data"


def tidy_data(interval=1):
    data = glob.glob("./result_data/*.csv")
    df = DataFrame(np.array(pd.read_csv(data[0],
header=None).values).reshape((240,1)), columns=['label'])
    for i, file in enumerate(data):
        if i == 0:
            continue
        else:
            df_f = DataFrame(np.array(pd.read_csv(data[i],
header=None).values).reshape((240,1)))
            df["{0} h".format(interval*(i-1))] = df_f
    df.dropna(how="any")
    return df


df = tidy_data(interval=1)
df = df.dropna(how="any")
df.to_csv("./result_data/data.csv")
```
